# Antibodies to unknown antigens other than swine leukocyte antigens on GTKO/β4GalNT2KO pig cells are associated with AHXR after pig-to-rhesus monkey kidney transplantation

**DOI:** 10.3389/fimmu.2025.1663702

**Published:** 2025-10-09

**Authors:** Hongtao Jiang, Haiyun Jiang, Songzhe He, Yuxiang Chen, Jiaxiang Du, Dengke Pan, Tao Li, Yi Wang

**Affiliations:** ^1^ Department of Kidney Transplantation, The Second Affiliated Hospital of Hainan Medical University, Haikou, China; ^2^ The Transplantation Institute of Hainan Medical University, Haikou, China; ^3^ Chengdu Clonorgan Biotechnology Co., Ltd., Chengdu, China

**Keywords:** antibodies, kidney, pig, genetically-modified, xenoantigen, xenotransplantation

## Abstract

**Background:**

Although progress in experimental life-supporting pig renal xenotransplantation has been encouraging, acute humoral xenograft rejection (AHXR) is still an obstacle to the survival of non-human primates that received genetically modified pig kidneys. This is possibly associated with the expression of xenoantigens in addition to the two known xenoantigens (Gal and Sda). We attempted to clarify the effect of elicited antibodies on GTKO/β4GalNT2KO-based pig-to-rhesus monkey renal xenotransplantation.

**Methods:**

Rhesus monkeys (n = 7) received kidneys from GTKO/β4GalNT2KO (n = 1) or GTKO/β4GalNT2KO/hCD55/hTBM (n = 3) pigs, and recipient serum was collected. Serum was incubated with GTKO/β4GalNT2KO pig red blood cells (pRBCs) to measure remaining antibodies to pig peripheral blood mononuclear cells (pPBMCs). Antibody binding and cytotoxicity of serum (either adsorbed on pig RBCs or unabsorbed) to GTKO/β4GalNT2KO or GTKO/β4GalNT2KO/hCD55/hTBM pig PBMCs or RBCs were measured by flow cytometry. At biopsy or euthanasia, the grafts were examined by histological assessment.

**Results:**

Survival of the seven recipients was <30 days. Serum creatinine was increased, and platelet count was decreased. Anti-pig antibodies (IgG or IgM) were elevated in the serum of all seven recipients at some time point. Histopathology of the kidneys showed features of AHXR and thrombotic microangiopathy in all grafts. Immunohistochemistry showed C3c, C4d, IgM and/or IgG, and C5b-9 deposition and CD68 infiltration in most grafts. Serum anti-pig antibodies remained elevated even after absorption on pig RBCs, which indicated that another xenoantigen, e.g., swine leukocyte antigens (SLAs) or “neoantigen I” (which also expresses on pig PBMCs but does not express on pig RBCs), may be playing a role in AHXR. Neoantigen I is an unidentified xenoantigen expressed on PBMCs, shared with kidney xenografts but not present on RBCs. There was a difference in antibody binding to PBMCs between unabsorbed and absorbed serum, suggesting the presence of anti-”neoantigen II” (which is expressed on pig RBCs and PBMCs) antibodies on PBMCs, which may be important in causing AHXR.

**Conclusions:**

These data suggest that elicited antibodies to “neoantigens”, e.g., non-SLA, play a role in AHXR after GTKO/β4GalNT2KO-based pig kidney transplantation in non-human primates.

## Introduction

End-stage renal disease is the outcome of various kidney diseases. Kidney transplantation is the optimal treatment for this condition, but the scarcity of human donors hinders its effectiveness. Despite the utilization of an expanded donor pool, the shortage of donor organs persists. As an alternative approach, kidney xenotransplantation emerges as a potential solution to address the challenges associated with deceased human donation.

In recent years, xenotransplantation has made great progress, largely through the development of genetically modified pigs as sources of organs (“donors”) (1–6). The longest published survival of a life-supporting pig kidney xenotransplant in a non-human primate (NHP) was 758 days (7). These results are encouraging, but many recipients survive for shorter periods of time, even when the preferred GTKO/β4GalNT2KO pigs are the donors, but only a few NHP recipients have received GTKO/β4GalNT2KO pig kidneys (8, 9). Antibody-mediated rejection developed within 3 months or 1 year in 42% and 46% recipients, respectively, with cellular rejection developing in only 3% and 3% recipients, respectively (10). These results strongly suggest that, in addition to the known xenoantigens (**Table 1**), there are other swine leukocyte antigens (SLAs) or possibly unknown xenoantigens with significant immunogenicity. However, there is still no consensus on whether SLA should be knocked out.

**Table 1:** Known carbohydrate xenoantigens expressed on pig cells.

Carbohydrate (abbreviation)	Responsible enzyme	Gene-knockout pig
1. Galactose-α1,3-galactose (Gal)	α1,3-Galactosyltransferase	GTKO
2. *N*-Glycolylneuraminic acid (Neu5Gc)	CMAH	CMAH-KO
3. Sd^a^	β-1,4-*N*-Acetylgalactosaminyltransferase	β4GalNT2KO

CMAH, cytidine monophosphate-*N*-acetylneuraminic acid hydroxylase.

SLA is among these potential xenoantigens, but whether it is preferable to eliminate the expression of SLA in pigs remains uncertain. When Adams et al. used GTKO/β4GalNT2KO/SLA-I KO as donor pigs, the longest and shortest survival periods of the recipient monkeys were 414 and 6 days, respectively (8), which suggests that other “neoantigens” may have immunogenicity in addition to SLA class I. To compare the immunogenicity strength of SLA and other xenoantigens, we defined unknown neoantigens (“neoantigen I” and “neoantigen II”) according to their different expression on pig red blood cells (pRBCs) and pig peripheral blood mononuclear cells (pPBMCs). “Neoantigen I” is expressed on PBMCs but not RBCs (e.g., carbohydrate but not SLA class I/II), and “neoantigen II” is expressed on both RBCs and PBMCs (e.g., carbohydrate).

The aim of the present study was to measure the levels of serum antibodies to unknown xenoantigens (which we have termed neoantigens, which may include SLA) after genetically modified pig-to-rhesus monkey renal transplantation when acute humoral xenograft rejection (AHXR) developed. To carry out these studies, we incubated monkey serum with pRBCs (which do *not* express SLA and “neoantigen I”) to absorb all other antibodies, allowing us to determine the presence of antibodies against “neoantigen II” expressed on PBMCs.

## Materials and methods

### Pig-to-rhesus monkey renal xenotransplantation

#### Donor pigs

Genetically modified pigs [GTKO/β4GalNT2KO (n = 1) or GTKO/β4GalNT2KO/hCD55/hTBM (n = 3)] were provided by Chengdu Clonorgan Biotechnology (Chengdu, Sichuan, China) (**Table 1**). These pigs were not raised in a Defined Pathogen-Free (DPF) environment, nor were they tested for cytomegalovirus expression.

#### Recipient monkeys

Rhesus monkeys (n = 7) from Hubei Tianqin Biotechnology Corporation, South China Primate Research Center (Hubei, China), and Clinical Immunology Translational Medicine Key Laboratory of Sichuan Province, Sichuan Academy of Medical Sciences & Sichuan Provincial People’s Hospital (Chengdu, China) weighed 6–11 kg. All recipients were selected on the basis of having low anti-pig antibody levels. The immunosuppressive (IS) regimens are shown in **Table 2**.

**Table 2:** Genetic modifications of donor pigs and survival in rhesus monkeys.

Donor pig	Recipient rhesus monkey			
Gene phenotype	Number	IS induction therapy	IS maintenance therapy	Survival (days)
GTKO/β4GalNT2KO	162667	rATG (25 mg, D0, D1, D2, D3) CD20 (100 mg, D−6) MP (10 mg/kg, D0, D1, D2)	*T, M (250 mg/d) P (5 mg/d) CD154 (20 mg/kg, D0, weekly)	16
GTKO/β4GalNT2KO/hCD55/hTBM	382409	rATG (25 mg, D0, D1, D2, D3) CD20 (100 mg, D−6) MP (10 mg/kg, D0, D1, D2)	*T, M (250 mg/d) P (5 mg/d) CD154 (20 mg/kg, D0, weekly)	24
	382292	CD4 (50 mg/kg, D−3, D0) CD20 (100 mg, D−6) MP (10 mg/kg, D0, D1, D2)	*T, M (250 mg/d) P (5 mg/d) CD154 (20 mg/kg, D0, weekly)	24
GTKO/β4GalNT2KO/hCD55/hTBM	061991	CD4 (50 mg/kg, D−3, D0) CD20 (100 mg, D−6) MP (10 mg/kg, D0, D1, D2)	*T, M (250 mg/d) P (5 mg/d) CD154 (20 mg/kg, D0, weekly)	14
	061997	rATG (25 mg, D0, D1, D2, D3) CD20 (100 mg, D−6) MP (10 mg/kg, D0, D1, D2)	*T, M (250 mg/d) P (5 mg/d) CD154 (20 mg/kg, D0, weekly)	14
GTKO/β4GalNT2KO/hCD55/hTBM	382205	ATG (25 mg, D0, D1, D2, D3) CD38 (100 mg, D−21, weekly) MP (10 mg/kg, D0, D1, D2)	*CsA, *Rapa CD38 (100 mg, weekly) M (250 mg/d) P (5 mg/d)	17
	823838	ATG (25 mg, D0, D1, D2, D3) CD38 (100 mg, D−21, weekly) MP (10 mg/kg, D0, D1, D2)	*CsA, *Rapa CD38 (100 mg, weekly) M (250 mg/d) P (5 mg/d)	29

CD154, anti-CD154mAb (NIH, USA); CD4, anti-CD4mAb (NIH, USA); CD38, anti-CD38mAb (NIH, USA); CD20, anti-CD20mAb (Roche Pharmaceutical Co., Ltd., Shanghai, China); hCD55, transgenic expression of the human complement-regulatory protein, CD55; CsA, cyclosporin A (Novartis, Beijing, China); hTBM, transgenic expression of human thrombomodulin; IS, immunosuppressive therapy; M, mycophenolate mofetil (Roche Pharmaceutical Co., Ltd., Shanghai, China); MP, methylprednisolone (Pfizer Manufacturing Belgium NV, Shenyang, China), P, prednisone (Tianjin Lisheng Pharmaceutical Company, Tianjin, China); Rapa, rapamycin (Fushen Biotechnology, Shanghai, China), rATG, rabbit thymoglobulin (Sanofi Pharmaceutical Co., Ltd., Beijing, China); T, tacrolimus (Astellas Pharmaceutical Co., Ltd., Liaoning, China.

*Tacrolimus (target 8–12 ng/mL), CsA (C0 target 200–400 ng/mL), and Rapa (target 8–12 ng/mL).

Animal care was in accordance with the Guide for the Care and Use of Laboratory Animals and approved by the Institutional Animal Care and Use Committee (IACUC) at Sichuan Provincial People’s Hospital and Tongji Hospital, Tongji Medical College, Huazhong University of Science and Technology; the work has been reported in accordance with the Animals in Research: Reporting of *In Vivo* Experiments (ARRIVE) guidelines (11).

#### Surgical procedures

##### Donor kidney extraction surgery

Anesthesia was induced with approximately 2 mL of Zoletil, and after endotracheal intubation, anesthesia was maintained with propofol. The donor pig was placed in a supine position, and a midline abdominal incision was made, sequentially cutting through the skin, subcutaneous tissue, and muscle layers. The peritoneum was opened, and the intestines were pushed to the right side and protected with warm, moist gauze. The retroperitoneum was opened to expose the left kidney. After dissecting the kidney and the entire ureter, the left renal vein was carefully freed up to the inferior vena cava, and the left renal artery was freed up to the abdominal aorta. After systemic heparinization of the donor pig, the donor kidney was excised and perfused *ex vivo* with 4°C histidine-tryptophan-ketoglutarate solution (HTK) solution until the kidney turned pale and the venous outflow became clear. The perfused kidney was preserved in a 4°C HTK solution for later use. The same procedure was applied to the right donor kidney. Finally, the donor pig was euthanized with intravenous injection of 10% potassium chloride (1 mL/kg) under deep anesthesia. After suturing the incision layer by layer, the body was discarded in a designated animal carcass disposal refrigerator. If only one kidney was harvested from the pig, the animal was returned to the animal room for continued housing.

##### Kidney transplantation surgery

Anesthesia was induced in the recipient monkey with approximately 0.5 mL of Zoletil, and after endotracheal intubation, anesthesia was maintained with propofol. The monkey was placed in a horizontal supine position, and the skin, subcutaneous tissue, and muscle layers were sequentially incised. The peritoneum was opened. The left peritoneum was opened, and the left naïve kidney and ureter were removed. The lower segment of the recipient monkey’s abdominal aorta and inferior vena cava were dissected. Under a surgical microscope, the donor pig’s renal artery and vein were anastomosed end-to-side to the abdominal aorta and inferior vena cava, respectively (using 8–0 suture for the artery and 7–0 suture for the vein). After the graft was perfused, the graft appeared ruddy in color and produced urine. Then, the donor ureter was anastomosed to the bladder (using 7–0 suture), and the recipient monkey’s right native kidney was removed. The incision was sutured layer by layer.

### Histological assessment of grafts

All specimens were examined by routine staining, immunofluorescence staining, and immunohistochemical staining.

### Rhesus monkey serum

Routinely, blood was drawn from the rhesus monkey (n = 7) before surgery and on days 1, 3, 7, 14, 21, and 28 after surgery, as well as under special circumstances (such as elevated creatinine levels). Blood collected in non-anticoagulant tubes (red tubes) was used for biochemical testing and serum extraction, while blood collected in anticoagulant tubes (purple tubes) was used for complete blood count testing. The serum was stored either as single or pooled samples at −80°C.

### Sources of pig cells

Whole blood was obtained from GTKO/β4GalNT2KO and GTKO/β4GalNT2KO/hCD55/hTBM pigs (Chengdu Clonorgan Biotechnology) in an anticoagulant tube (green tube), and PBMCs and RBCs were isolated, as previously described (12–14).

### Absorption of monkey serum anti-pig antibodies on pRBCs

Serum samples obtained from seven recipient subjects at various time points were employed. GTKO/β4GalNT2KO RBCs (>1 × 10^8^) were added to an Eppendorf (EP) tube and centrifuged at 8,000 rpm for 10 min at room temperature. After carefully aspirating the supernatant, 100 μL of monkey serum was added to the pRBCs at room temperature after continuous mixing in a 360° mixer for 1 hour. The EP tube was centrifuged at 8,000 rpm for 10 min at room temperature. The supernatant was carefully aspirated and transferred into a new EP tube.

### Binding of serum IgM and IgG to pRBCs and pPBMCs by flow cytometry

Using the adsorbed serum samples, binding of serum antibodies to pig cells was measured by flow cytometry using the relative geometric mean (rGM), as previously described (14).

### Serum complement-dependent cytotoxicity of pPBMCs by flow cytometry

Using the adsorbed serum samples, briefly, PBMCs (5 × 10^5^ cells in 250 µL FACS buffer) were incubated with 50 µL heat-inactivated recipients’ serum at 4°C for 1 hour. After washing with phosphate-buffered saline (PBS), fluorescence-activated cell sorting (FACS) buffer (200 µL) and rabbit complement (50 µL, Cedarlane, Hornby, CA, USA) were added (final concentration 20%), and incubation was carried out at 37°C for 30 min. After washing with PBS, the cells were incubated in the dark at 4°C for 15 min with propidium iodide, and finally, 200 µL FACS buffer was added. Flow cytometry was carried out using BD FACSCelesta.

Cytotoxicity was calculated as follows:


$$\%\text{ cytotoxicity}=\left(\left[\text{A}-\text{C}\right]/\left[\text{B}-\text{C}\right]\right)\times 100,$$

where A represents the percentage of dead cells, B is the maximal percentage of dead cells (PBMCs fixed with 70% ethanol), and C is the minimal percentage of dead cells (PBMCs incubated with rabbit complement only) (15).

### Statistical analysis

Significance of the difference between two groups was determined using Student’s t-test or the Wilcoxon test. Continuous variables were expressed as mean ± SD. Comparisons among multiple groups were performed using a one-way ANOVA test (Tukey’s test) or a non-parametric test (Dunn’s test). A p-value of <0.05 was considered statistically significant. All statistical analyses were performed using social science software GraphPad Prism 8 (GraphPad Software, San Diego, CA, USA).

## Results

### Survival of recipient monkeys and histopathological findings of grafts

**Figure 1:**
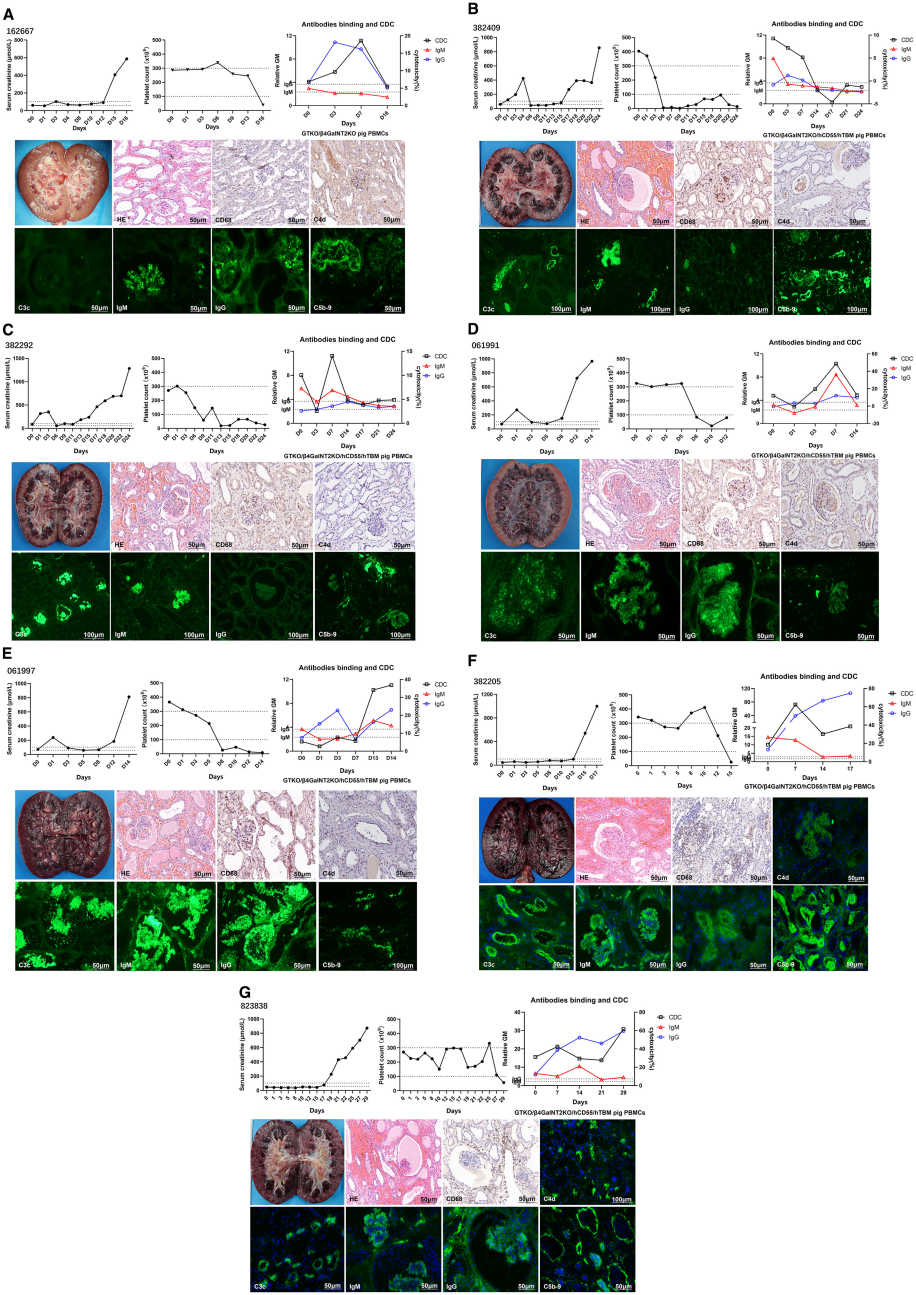
Monitoring of recipient monkeys throughout the course of the experiment and microscopic appearances of the pig kidney grafts at biopsy on the day they were euthanized. **(A)** Samples from monkey 162667. serum creatinine (sCr) levels, platelet counts, anti-pig IgM/IgG levels, and CDC following kidney xenotransplantation. Gross finding of hemorrhagic kidney at POD 16. H&E showed mild interstitial hemorrhage and tubular necrosis. Immunohistochemical picture of CD68 showed mild infiltration, and C4d showed mild deposition. Immunofluorescence pictures of IgM, IgG, and C5b-9 showed deposition, except for C3c. **(B)** Samples from monkey 382409. sCr levels, platelet counts, anti-pig IgM/IgG levels, and CDC following KXTx. Gross finding of hemorrhagic kidney at POD 24. H&E showed great interstitial hemorrhage, microcirculatory thrombosis, and tubular necrosis. Immunohistochemical picture of CD68 showed great infiltration, and C4d showed mild deposition. Immunofluorescence pictures of C3c, IgM, IgG, and C5b-9 showed deposition. **(C)** Samples from monkey 382292. sCr levels, platelet counts, anti-pig IgM/IgG levels, and CDC following KXTx. Gross finding of hemorrhagic kidney at POD 24. H&E showed great interstitial hemorrhage and tubular necrosis. Immunohistochemical picture of CD68 showed great infiltration, and C4d showed mild deposition. Immunofluorescence pictures of C3c, IgM, and C5b-9 showed deposition, except for IgG. **(D)** Samples from monkey 061991. sCr levels, platelet counts, anti-pig IgM/IgG levels, and CDC following KXTx. Gross finding of hemorrhagic kidney at POD 14. H&E showed great interstitial hemorrhage and tubular necrosis. Immunohistochemical picture of CD68 showed great infiltration, and C4d showed mild deposition. Immunofluorescence pictures of C3c, IgM, IgG, and C5b-9 showed deposition. **(E)** Samples from monkey 061997. sCr levels, platelet counts, anti-pig IgM/IgG levels, and CDC following KXTx. Gross finding of hemorrhagic kidney at POD 14. H&E showed great interstitial hemorrhage, microcirculatory thrombosis, and tubular necrosis. Immunohistochemical picture of CD68 showed great infiltration, and C4d showed mild deposition. Immunofluorescence pictures of C3c, IgM, IgG, and C5b-9 showed deposition. **(F)** Samples from monkey 382205. sCr levels, platelet counts, anti-pig IgM/IgG levels, and CDC following KXTx. Gross finding of hemorrhagic kidney at POD 17. H&E showed great interstitial hemorrhage, microcirculatory thrombosis, and tubular necrosis. Immunohistochemical picture of CD68 showed great infiltration. Immunofluorescence pictures of C4d, C3c, IgM, IgG, and C5b-9 showed deposition. **(G)** Samples from monkey 823838. sCr levels, platelet counts, anti-pig IgM/IgG levels, and CDC following KXTx. Gross finding of hemorrhagic kidney at POD 29. H&E showed great interstitial hemorrhage, microcirculatory thrombosis, and tubular necrosis. Immunohistochemical picture of CD68 showed great infiltration. Immunofluorescence pictures of C4d, C3c, IgM, IgG, and C5b-9 showed deposition. CDC, complement-dependent cytotoxicity.

We performed seven genetically modified pig-to-monkey kidney transplants. All subjects developed AHXR and were euthanized within 30 days, and most of the grafts showed hemorrhage. H&E of grafts showed interstitial hemorrhage, microcirculation thrombus, and tubular necrosis. Immunohistochemistry and immunofluorescence of most grafts showed CD68 infiltration and C4d, C3c, IgM, IgG, and C5b-9 deposition (**Figures 1A–G**).

When the recipient’s platelet count decreased, thrombopoietin receptor agonists (TPO-RAs) failed to elevate platelet levels. The primary reason was related to microthrombus formation in the transplanted kidney’s microcirculation, which consumes platelets. We observed that the rise in the recipient’s serum creatinine mostly occurred later than the decline in platelets. This may be because microthrombi had already formed in the porcine kidney’s microcirculation at an early stage, consuming platelets. However, due to the large size of the porcine kidney, even if some renal tissue was damaged, it did not immediately lead to a rise in creatinine. As the damage to the transplanted kidney worsened, it eventually became unable to meet the recipient’s metabolic demands, leading to the observed increase in creatinine. Therefore, a decrease in platelet count may serve as an early marker of transplanted kidney injury.

### Antibodies binding to SLA and/or “neoantigen I” in monkey recipients after pig kidney transplantation

The serum of the recipients was collected sequentially and stored at −80 °C. The serum was incubated with GTKO/β4GalNT2KO pRBCs, after which IgM/IgG binding and complement-dependent cytotoxicity (CDC) against RBCs or PBMCs from the same pigs were measured using flow cytometry.

**Figure 2:**
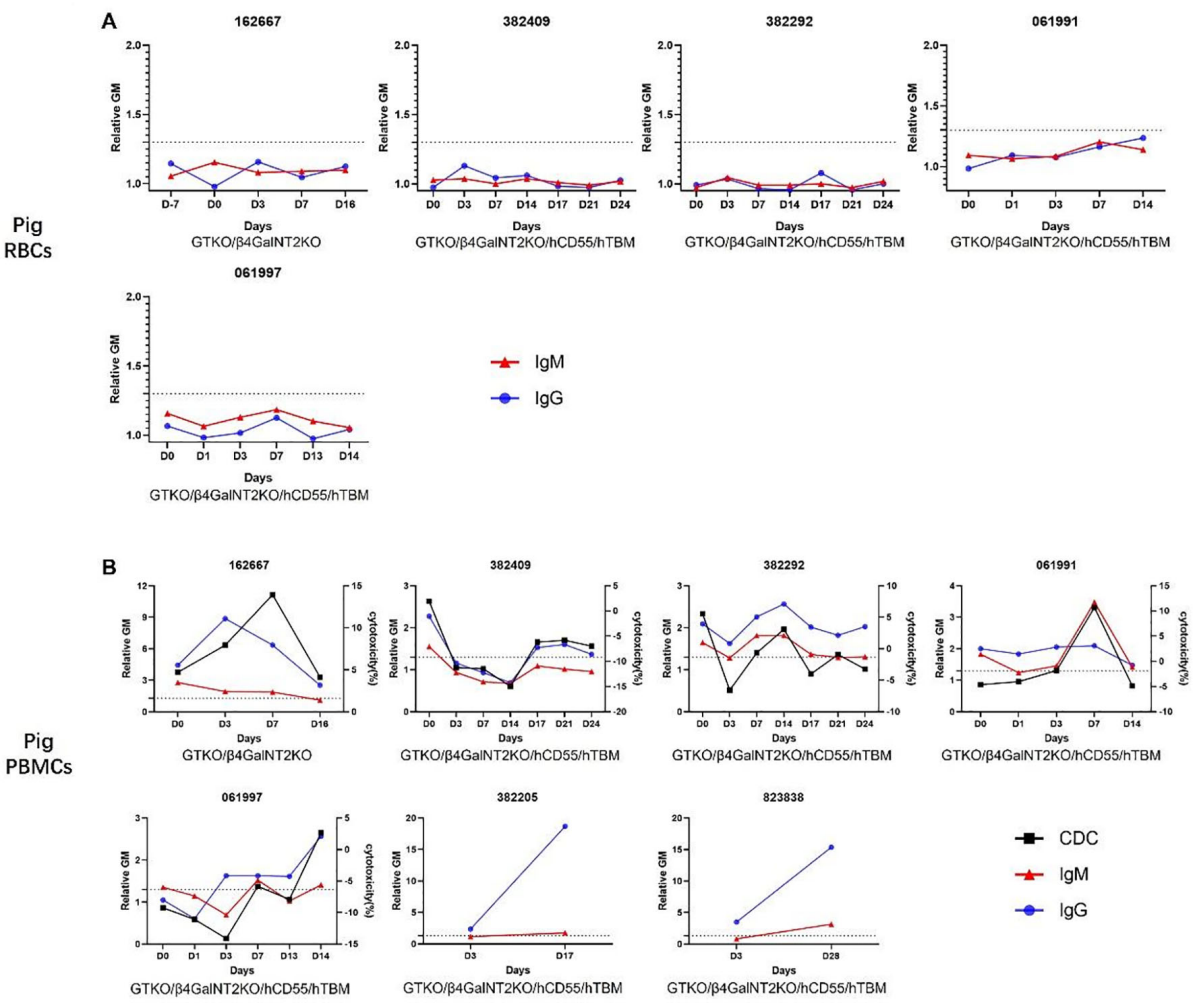
IgM and IgG binding and CDC to pRBCs and pPBMCs in the absorbed sera of monkeys with pig kidney grafts. **(A)** There was *no* detectable binding of IgM and IgG to GTKO/β4GalNT2KO or GTKO/β4GalNT2KO/hCD55/hTBM pRBCs throughout the post-transplant course. We did not test antibody binding in the last two sera because of a lack of samples. **(B)** There were increases and variations in IgM and IgG binding and CDC to GTKO/β4GalNT2KO or GTKO/β4GalNT2KO/hCD55/hTBM PBMCs throughout the post-transplant course. Blood from 382205 and 823838 was drawn only twice, and CDC was not measured during the post-transplant course. CDC, complement-dependent cytotoxicity; pRBCs, pig red blood cells; pPBMCs, pig peripheral blood mononuclear cells.

After incubation, serum IgM/IgG binding to pRBCs was negative at each time point in all recipient monkeys (**Figure 2A**), indicating that serum anti-”neoantigen II” antibodies had been completely absorbed by GTKO/β4GalNT2KO pRBCs (except possibly anti-SLA antibodies and antibodies to “neoantigen I”).

pRBC-absorbed serum IgM or IgG in all recipients showed binding to GTKO/β4GalNT2KO or GTKO/β4GalNT2KO/hCD55/hTBM PBMCs *before* transplantation (**Figure 2B**), although in one case, binding was minimal (061997), indicating that the serum of recipients included preformed anti-SLA and/or anti-”neoantigen I” antibodies. Serum IgM or IgG binding to pPBMCs was increased at some time points *after* transplantation. The data suggested that the development of elicited antibodies to “neoantigens” (possibly SLA or “neoantigen I”) had initiated AHXR.

### Antibodies bind to “neoantigen II” more than to SLA and/or “neoantigen I” in most monkey recipients after pig kidney transplantation

**Figure 3:**
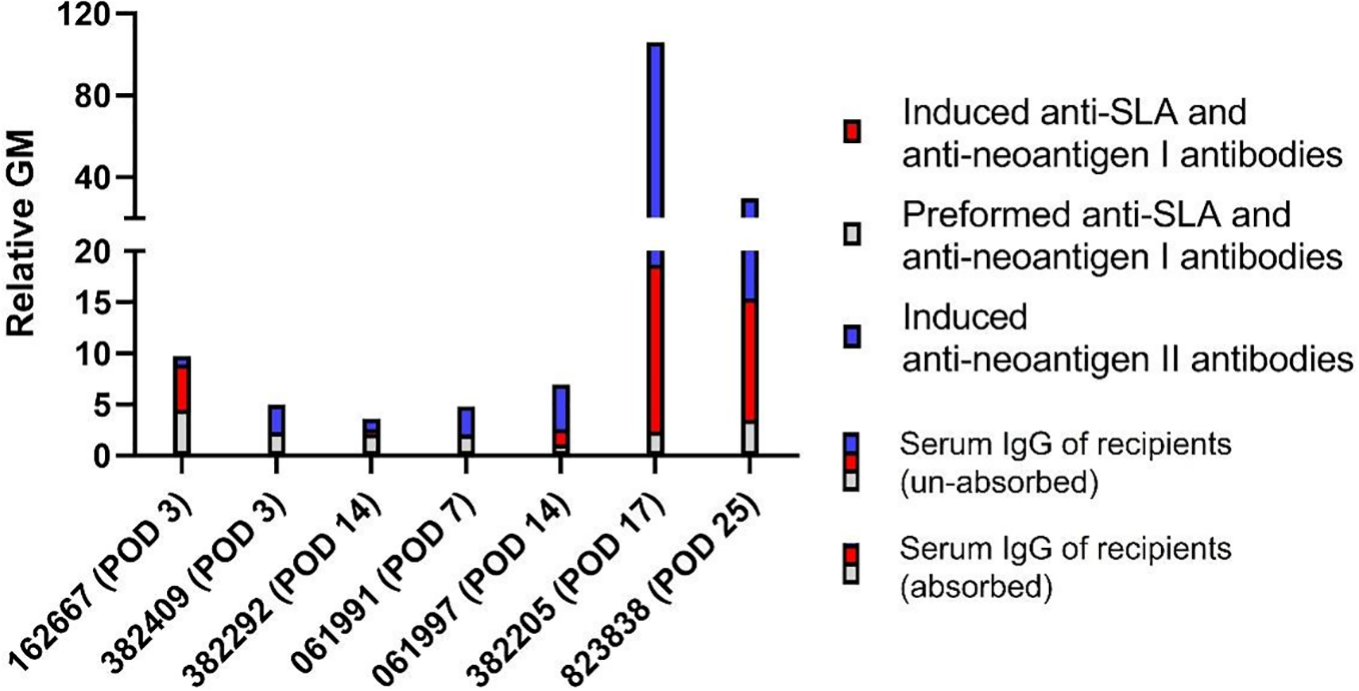
IgG binding to pPBMCs in the absorbed and unabsorbed sera of monkeys with pig kidney grafts. Recipients’ unabsorbed serum IgG (blue with red with gray) or absorbed serum IgG (red with gray) binding to GTKO/β4GalNT2KO or GTKO/β4GalNT2KO/hCD55/hTBM PBMCs post-transplant. Recipients’ absorbed serum IgG binding to GTKO/β4GalNT2KO or GTKO/β4GalNT2KO/hCD55/hTBM PBMCs pre-transplant (gray). The difference in absorbed serum IgG binding to pPBMCs post-transplant and absorbed serum IgG binding to pPBMCs pre-transplant (red). The difference in unabsorbed serum IgG binding to pPBMCs post-transplant and unabsorbed serum IgG binding to pPBMCs post-transplant (blue). pPBMCs, pig peripheral blood mononuclear cells.

Considering the variation in serum IgG levels among recipients in **Figure 1**, we chose the specific time point corresponding to the peak increase in serum IgG following transplantation [**Figure 3**, serum IgG of recipients (unabsorbed)]. Meanwhile, we selected serum IgG of recipients on the same day [**Figure 3**, serum IgG of recipients (absorbed)] and pre-operation day (**Figure 3**, preformed anti-SLA and anti-neoantigen I antibodies) as shown in **Figure 2**. Specifically, for recipients 162667, 382409, 382292, 061991, 061997, 382205, and 823838, we selected D3, D3, D14, D7, D14, D17, and D25, respectively. From these results, serum IgG that increased in recipient 162667 on the POD 3 was mainly composed of induced anti-SLA and anti-”neoantigen I” antibodies (blue vs. red: 0.84 vs. 4.43). The other six recipients had elevated serum IgG, mainly composed of induced anti-”neoantigen II” antibodies on POD 3 (382409 blue vs. red: 2.69 vs. 0), POD 14 (382292 blue vs. red: 1.01 vs. 0.48), POD 7 (061991 blue vs. red: 2.71 vs. 0.10), POD 14 (061997 blue vs. red: 4.35 vs. 1.51), POD 17 (382205 blue vs. red: 87.28 vs. 16.33), and POD 25 (823838 blue vs. red: 14.37 vs. 11.88). These results suggest that even if SLA is knocked out, the unknown “neoantigen II” can cause recipients to develop AHXR.

## Discussion

Renal xenotransplantation has made considerable progress, largely from the production of genetically modified pigs. However, many pig grafts are lost due to AHXR and/or thrombotic microangiopathy (8, 9, 16–19). AHXR more commonly occurs as an acute rejection process, generally emerging within weeks to months after transplantation. The diagnosis and monitoring of antibody-mediated rejection (AMR) in these models rely on several key biological biomarkers, many of which hold significant clinical relevance. Important serological indicators include elevated titers of anti-non-Gal antibodies, increased levels of total IgG and IgM, and a rise in anti-pig antibody levels. Histopathological assessment remains a cornerstone for confirming AMR. Characteristic findings include thrombotic microangiopathy (TMA) and diffuse deposition of complement components C4d and/or C3d along the capillary endothelium, which serves as critical evidence of complement activation. Additionally, deposits of IgG and/or IgM can often be detected immunohistochemically, further supporting the diagnosis. We have previously demonstrated that anti-Sda antibodies were elevated in the serum of two recipients that received GTKO pig kidneys and developed AHXR (12). Sda antigens may therefore contribute to AHXR (12). Rather than generating a new pig, it is essential to prevent sensitization (i.e., the development of *de novo* antibodies) in recipients after xenotransplantation. Indeed, two out of seven recipients that were not treated with CD40–CD154 pathway blockade (i.e., anti-CD154 mAbs) showed increased levels of anti-pig antibodies (including neoantigens I and II and SLA) after transplantation compared to the recipients that received anti-CD154 mAbs. Unfortunately, sensitization could not be prevented in all recipients, even with the use of anti-CD154 mAbs. Therefore, modifying IS regimens is necessary for future studies. Meanwhile, these results suggest that, in addition to the known important xenoantigen, other neoantigens may induce AHXR.

Currently, at least three carbohydrate antigens (Gal, Neu5Gc, and Sda) are recognized as xenograft antigens in humans. Their removal from pig cells significantly diminishes human antibody binding and CDC. Notably, TKO pigs, engineered to lack these three xenoantigens, have been utilized as donor sources in two recent heart transplant cases involving living human recipients, as well as in preclinical studies with brain-dead patients.

Unlike humans, TKO pigs are unsuitable for organ xenografts into Old World Nonhuman Primates (OWNHPs). Instead, a double knockout pig (GTKO/β4GalNT2KO) is recommended as the optimal donor pig for OWNHPs. However, OWNHPs still possess naturally preformed antibodies against these pigs, leading to antibody-mediated xenograft rejection after transplantation (e.g., AHXR). Thus, selecting recipients with a low titer of anti-pig antibodies is crucial for the success of long-term xenograft survival.

Most researchers believe SLA to be a xenoantigen (20–23), but there are relatively few studies on the role of SLA in xenotransplantation (23–25). The model of GTKO-based or GTKO/β4GalNT2KO-based pig-to-Old World monkey (which expresses Neu5Gc antigens) transplantation is difficult when studying anti-SLA antibodies because of elevated levels of other anti-pig antibodies. Recipients still developed AHXR even though they received GTKO/β4GalNT2KO/SLA-IKO pig kidneys (8); the results suggested that neoantigen plays a crucial role in AHXR in addition to SLA. We defined “neoantigen I” and “neoantigen II” according to the difference in the expression of antigens on pRBCs and pPBMCs. Therefore, we were able to investigate the role of antibodies against unknown “neoantigens” by absorbing the monkey serum on pRBCs. Six of seven recipients that received GTKO/β4GalNT2KO-based pig kidneys developed anti-SLA and/or anti-”neoantigen I” antibodies after transplantation, according to our results, and IgG dominated in the elevated antibodies. One recipient (382409) had high levels of preformed anti-SLA and/or anti-”neoantigen I” antibodies. Whether AHXR was associated with the presence of preformed or elicited antibodies in this recipient remains uncertain, but both could have played a role. Although we have no definitive evidence from the present study, it is likely that SLA is associated with the observed increase in antibodies against neoantigens in the study. Li et al. confirmed that knockout of Gal, Neu5Gc, Sda, and SLA-I significantly reduced the immunogenicity of porcine cells, but still retained the ability to activate human NK cells (26). Hein et al. demonstrated that the lack of expression of SLA-I is an effective way to prevent the activation of human CD8^+^ T cells (20). The absence or lowered expression of SLA-II significantly reduced the proliferation of human CD4^+^ T cells (8, 27, 28). Therefore, SLA-KO may play a positive role in prolonging the survival of recipients of life-supporting pig grafts. However, we need to further investigate the role of SLA and “neoantigens (I and II)” in xenograft survival.

In our study, anti-pig IgG was elevated in the serum of all recipients after xenotransplantation. Interestingly, induced anti-”neoantigen II” antibodies dominated in six recipients, which means that recipient monkeys will still develop AHXR even when they received GTKO/β4GalNT2KO/SLA-KO pig kidneys. Therefore, identifying unknown neoantigens (especially expressed on both pPBMCs and pRBCs) is more important; liquid chromatography-mass spectrometry (LC-MS) and new experimental techniques in the future could help with this problem. Definitive identification of these neoantigens may lead to their knockout and thus improve the results of pig organ xenotransplantation.

In conclusion, our study demonstrated that i) the expression of SLA and/or unknown neoantigens can stimulate the generation of new antibodies to GTKO/β4GalNT2KO-based pig kidneys in rhesus monkeys, especially “neoantigen II”, which is expressed on both pPBMCs and pRBCs, and ii) these are associated with the development of AHXR.

## Data Availability

The original contributions presented in the study are included in the article/supplementary material. Further inquiries can be directed to the corresponding authors.
